# A case of low-grade endometrial stromal sarcoma presented as an intramyometrial mass mimicking uterine leiomyoma on MRI

**DOI:** 10.1093/bjrcr/uaad012

**Published:** 2023-12-18

**Authors:** Soichiro Tamada, Hiromi Edo, Taishi Sakima, Ryo Tanaka, Kohei Shikata, Soko Nishitani, Morikazu Miyamoto, Masashi Takano, Keisuke Kuboshima, Kosuke Miyai, Sho Ogata, Hiroshi Shinmoto

**Affiliations:** Department of Radiology, National Defense Medical College, Saitama 359-8513, Japan; Department of Radiology, National Defense Medical College, Saitama 359-8513, Japan; Department of Radiology, National Defense Medical College, Saitama 359-8513, Japan; Department of Radiology, National Defense Medical College, Saitama 359-8513, Japan; Department of Radiology, National Defense Medical College, Saitama 359-8513, Japan; Department of Obstetrics and Gynecology, National Defense Medical College, Saitama 359-8513, Japan; Department of Obstetrics and Gynecology, National Defense Medical College, Saitama 359-8513, Japan; Department of Obstetrics and Gynecology, National Defense Medical College, Saitama 359-8513, Japan; Department of Pathology and Laboratory Medicine, National Defense Medical College Hospital, Saitama 359-8513, Japan; Department of Pathology and Laboratory Medicine, National Defense Medical College Hospital, Saitama 359-8513, Japan; Department of Pathology and Laboratory Medicine, National Defense Medical College Hospital, Saitama 359-8513, Japan; Department of Radiology, National Defense Medical College, Saitama 359-8513, Japan

**Keywords:** low-grade endometrial stromal sarcoma, leiomyoma, MRI

## Abstract

A low-grade endometrial stromal sarcoma (ESS) has a pattern of presenting as an intramyometrial mass and is often misdiagnosed as cellular leiomyoma or degenerative uterine leiomyoma. A low-grade ESS is a malignant tumour that requires total hysterectomy with bilateral salpingo-oophorectomy; while a leiomyoma is a benign tumour and could be acceptable for enucleation. As the treatment strategies differ between a low-grade ESS and leiomyoma, radiologists should be familiar with the characteristic MRI findings of a low-grade ESS. A 51-year-old woman with abnormal uterine bleeding had been observed for 2 years at a previous hospital for a uterine leiomyoma based on MRI findings. A contrast-enhanced MRI demonstrated an intramyometrial mass composed of three components with the hypointense rim on T2-weighted images (T2WI): the first component was a homogeneous solid structure with mild hyperintensity on T2WI with a low apparent diffusion coefficient value; the second component was cystic; the third component was a structure of low signal intensity on T2WI similar to the muscle. Although a degenerative uterine leiomyoma was a differential diagnosis, these MRI findings were suggestive of a low-grade ESS. A total abdominal hysterectomy, bilateral salpingo-oophorectomy, pelvic lymphadenectomy, and partial omentectomy were performed. The pathological diagnosis was a low-grade ESS. In a low-grade ESS, there are three major patterns of MRI findings: one of these patterns is the less popular but clinically important intramyometrial mass pattern, which can be misdiagnosed as a leiomyoma, and this case conformed to this pattern.

## Clinical presentation

A 51-year-old woman with abnormal uterine bleeding had been observed for 2 years at a previous hospital for a uterine leiomyoma based on MRI findings. The patient was referred to our hospital due to the closing of the previous hospital.

## Investigations

**Figure 1.**
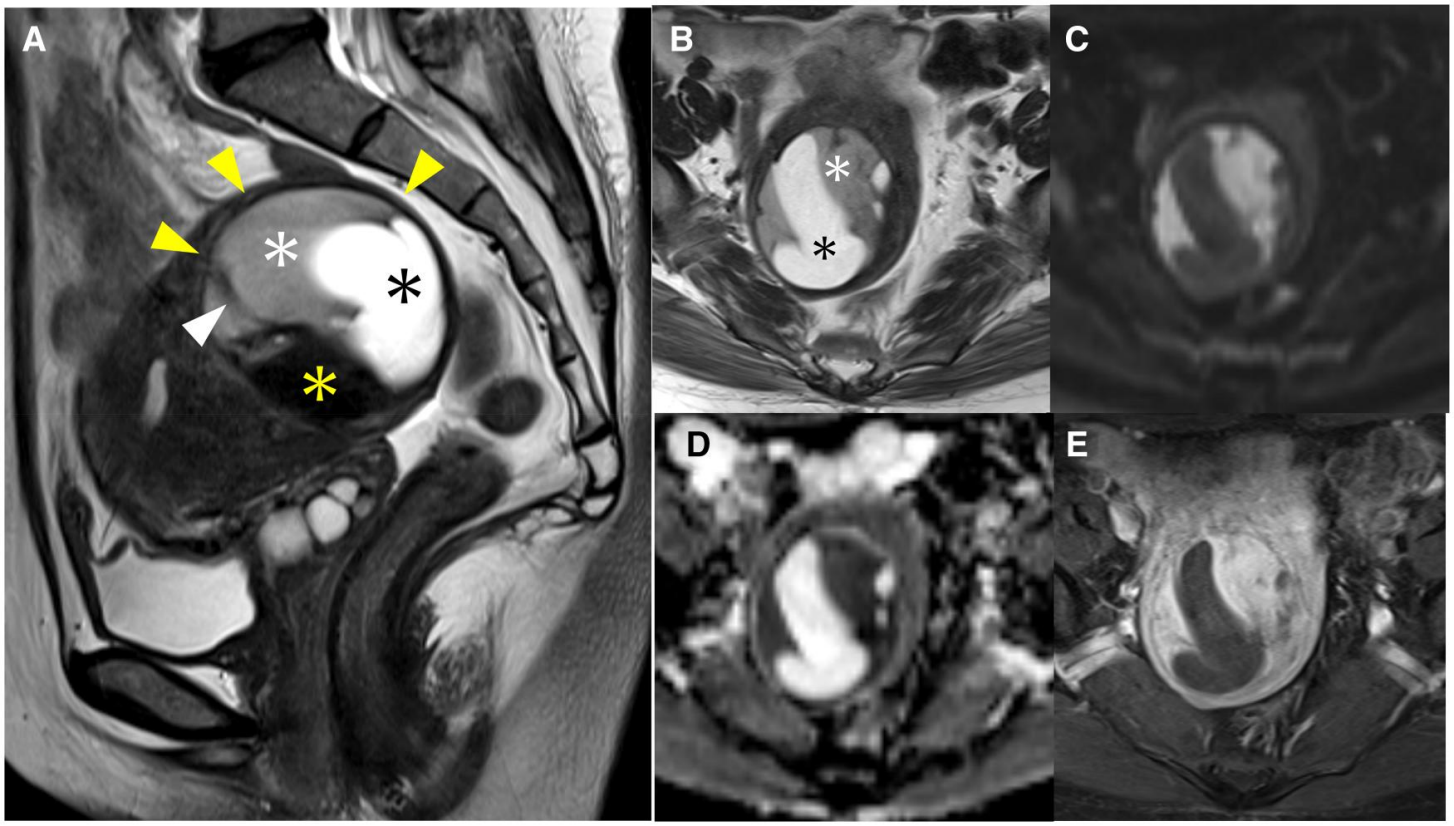
(A-E) Initial contrast-enhanced MRI performed 2 years prior at a previous hospital. A sagittal T2WI (A) showing a 6 cm mass located in the myometrium with a well-defined rim of low signal intensity (yellow arrowheads), consisting of three components: a homogeneous solid structure with mild hyperintensity (white asterisk), a cystic component (black asterisk), and a low-signal-intensity component similar to that of the muscle (yellow asterisk), along with a banded structure of low signal intensity (white arrowhead). An axial T2WI (B) showing a mass exhibiting a homogeneous solid component with mild hyperintensity (white asterisk) and a cystic component (black asterisk), accompanied by a low-signal-intensity rim surrounding the mass. The homogeneous solid component within the mass shows a high signal intensity on DWI (C), a low signal intensity on ADC map (D) with the ADC value of 1.08 × 10^−3^ mm^2^/s, and a slightly stronger enhancement than the myometrium on contrast-enhanced fat-suppressed T1WI (E).

**Figure 2.**
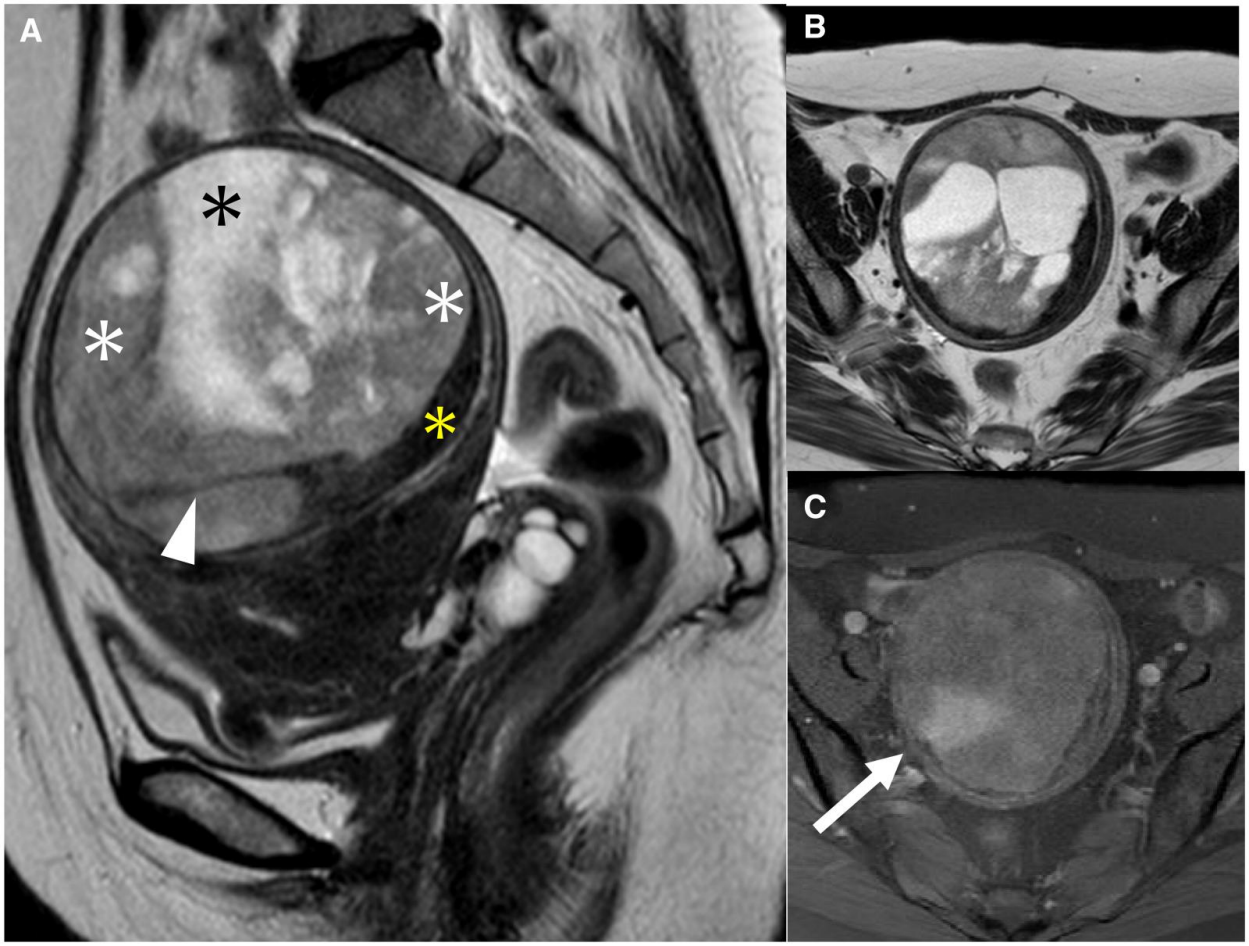
(A-C) The second contrast-enhanced MRI performed at the visit to our hospital. A sagittal T2WI (A) shows the mass enlarged to 9 cm compared to the initial MRI 2 years prior. In a sagittal T2WI (A), the homogeneous solid component with mild hyperintensity (white asterisk) and cystic component (black asterisk) are increased, the low-signal-intensity component similar to the muscle (yellow asterisk) is stretched, and the low signal intensity banded structure (white arrow) is not significantly changed compared to the initial MRI. The solid component with a moderately high signal intensity on T2WI (B) demonstrates a high signal intensity on fat-suppressed T1WI (C), suggesting of haemorrhage.

An initial contrast-enhanced MRI performed two years prior at the previous hospital showed an intramyometrial mass of 6 cm with a well-defined low-signal-intensity rim on T2WI (Figure 1A). The mass consisted of three different components: the first component was a homogeneous solid structure which showed a mild hyperintensity on T2WI and diffusion-weighted images (DWI) with a low apparent diffusion coefficient (ADC) value, which was enhanced slightly stronger than the myometrium on fat-suppressed T1-weighted images (T1WI); the second component was a cystic structure without haemorrhage; the third component showed a low signal intensity on T2WI, and a part of it showed a banded structure (Figure 1). A second contrast-enhanced MRI performed at our hospital showed that the mass had enlarged to 9 cm (Figure 2). The solid component with a mild hyperintensity on T2WI and the cystic component were enlarged, whereas the component that showed a low signal intensity on T2WI remained the same. Moreover, the fat-suppressed T1WI demonstrated a high signal intensity within the homogeneous solid component, suggesting of haemorrhage (Figure 2). No metastases were detected on the trunk CT.

## Differential diagnosis

In the initial MRI performed at the previous hospital, a well-defined tumour in the uterine myometrium was identified, which did not communicate with the endometrial cavity and showed cystic degeneration. The tumour demonstrated a moderately high signal intensity on T2WI and a high signal intensity on DWI, suggesting of a lesion with a high cellular density. The differential diagnoses included a low-grade endometrial stromal sarcoma (ESS) and cellular leiomyoma with degeneration. In the second MRI performed 2 years later, the tumour increased in size and showed haemorrhage within the solid component. The solid component, suggestive of a high cellular density, had also increased, leading to a stronger suspicion of a low-grade ESS than cellular leiomyoma with degeneration. Other differential diagnoses include smooth-muscle tumours of uncertain malignant potential (STUMP) and leiomyosarcoma.

## Treatment

**Figure 3.**
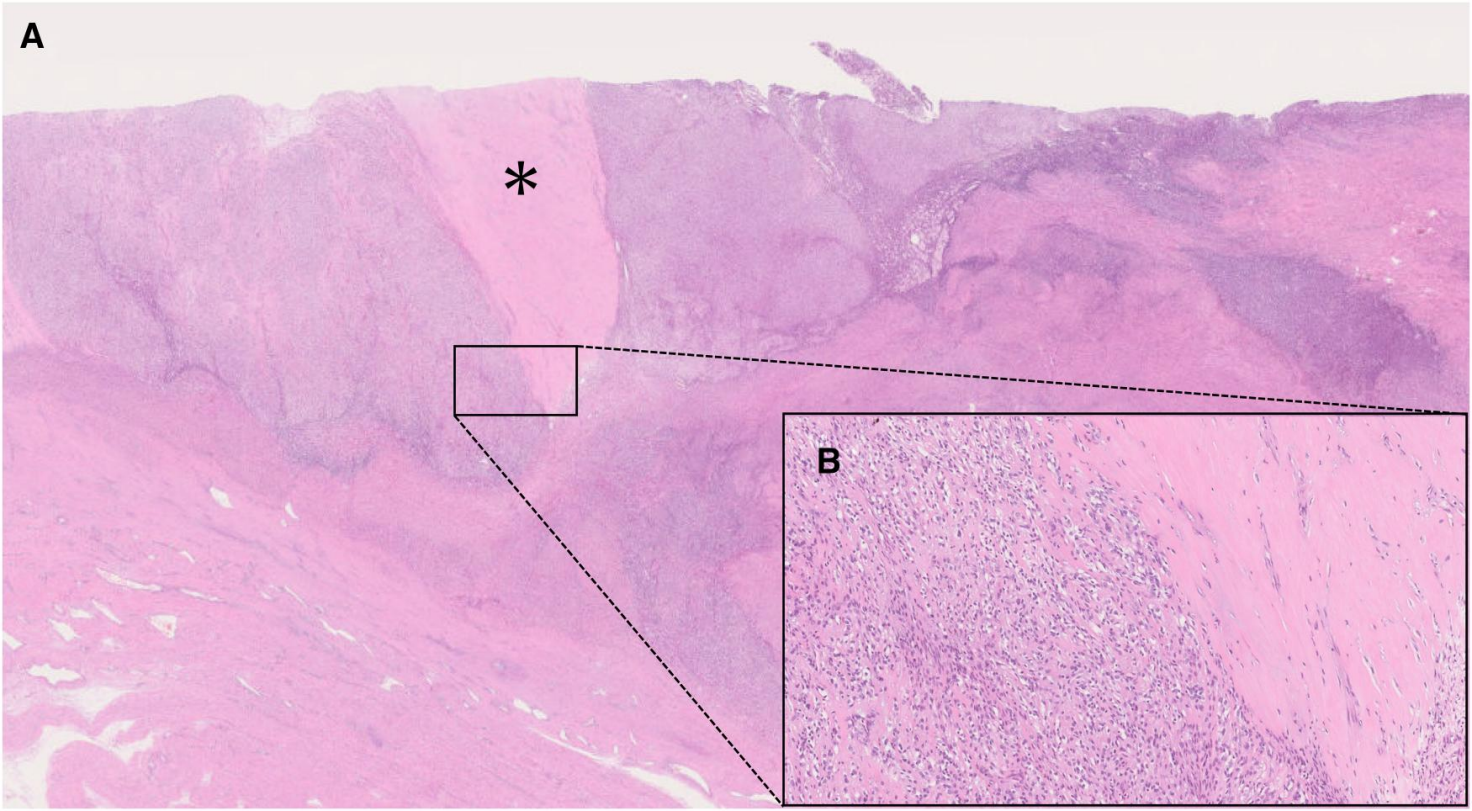
(A, B) Microscopic images. The black frame in (A) is enlarged as (B). The high-power field of haematoxylin-eosin staining shows that the tumour cells with low nuclear atypia infiltrate the myometrium (B). The banded myometrium (black asterisk) that has spared tumour cell invasion remains (A).

**Figure 4.**
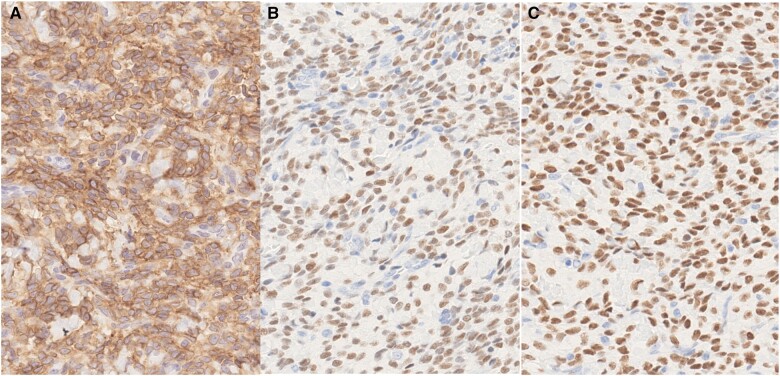
(A-C) In immunostaining, CD10 (A), ER (B), and PgR (C) are positive.

A total abdominal hysterectomy, bilateral salpingo-oophorectomy, pelvic lymphadenectomy, and partial omentectomy were performed. The histopathological examination showed that the tumour cells with low nuclear atypia infiltrated the myometrium (Figure 3), with the remaining areas of myometrium in the foci of tumour cells. The immunostaining was positive for CD10, ER, and PgR (Figure 4). The tumour was diagnosed as a low-grade ESS, and no lymph node metastasis or peritoneal dissemination was observed.

## Outcome and follow-up

No apparent recurrence or metastasis was detected 12 months after the surgery.

## Discussion

A low-grade ESS that does not communicate with the uterine cavity and presents as an intramyometrial tumour can easily be misdiagnosed as a uterine leiomyoma on MRI.^1–4^ If it is misdiagnosed as a uterine leiomyoma and an enucleation is performed, there is an increased risk of peritoneal dissemination and recurrence.^1^^,^^5^ Therefore, when an intramyometrial mass shows the characteristics of a low-grade ESS on MRI, radiologists should alert the gynaecologists of the possibility of a low-grade ESS.

A low-grade ESS is a rare uterine stromal tumour accounting for 0.2% of all uterine malignancies^6^ and is the second most common sarcoma occurring in the uterus. A low-grade ESS is a malignant stromal tumour comprising cells resembling the proliferative-phase of endometrial stroma and displays an infiltrative growth with or without a lymphovascular invasion.^7^

The MRI findings of a low-grade ESS have been described in three major patterns.^2^^,^^8^ The first pattern, which is widely recognized, is the progression of the tumour from the endometrium into the myometrium.^8^ The poorly known second pattern, is a well-defined mass located in the myometrium,^2^ as seen in the present case. The third pattern is a polypoid mass within the uterine cavity, which is very rare.^8^

**Figure 5.**
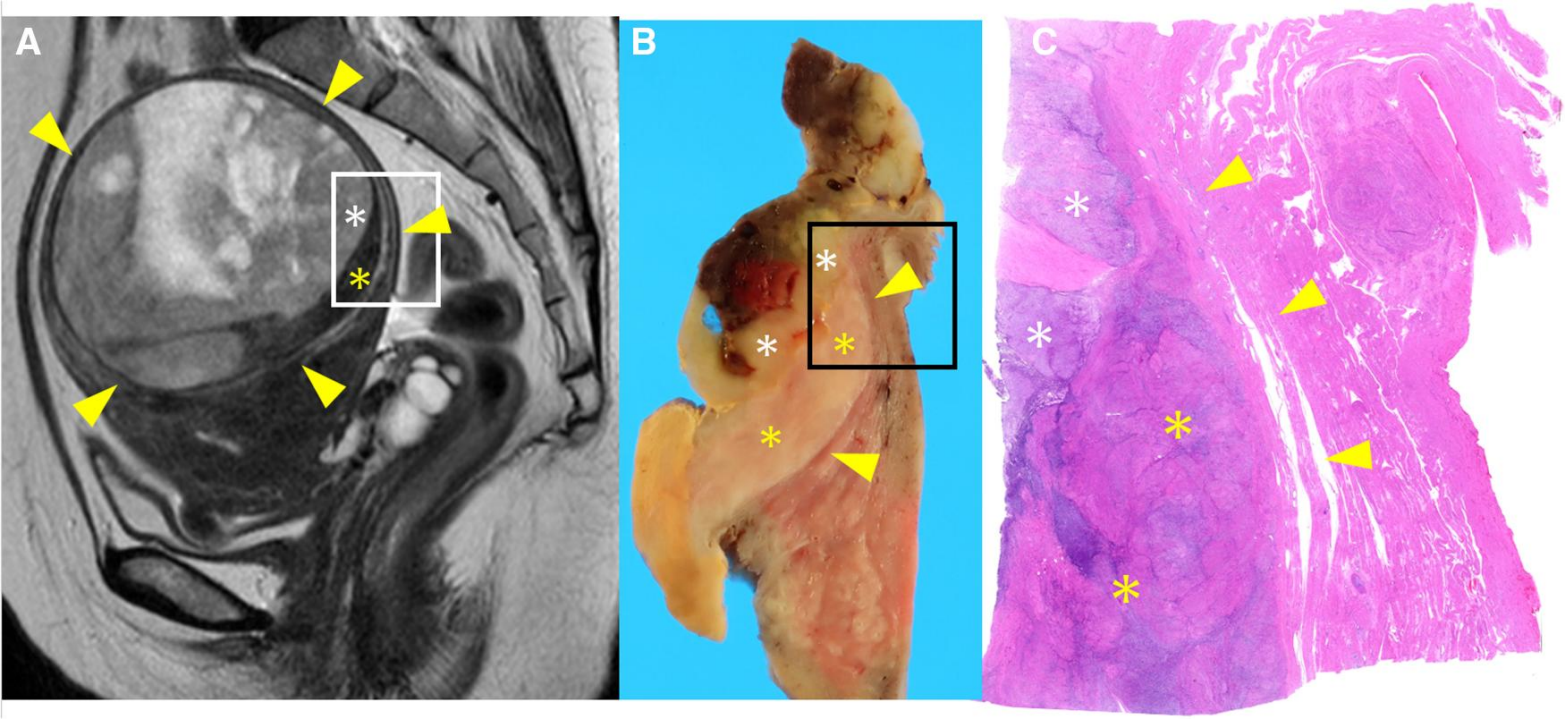
Sagittal T2WI (A), macroscopic image (B), and microscopic image (C). The homogeneous solid component with mild hyperintensity on T2WI (white asterisk) shows dense concentration of tumour cells in the microscopic view. The low-signal-intensity component on T2WI (yellow asterisk) shows a mixture of tumour cells and myometrium, with a relatively higher proportion of myometrium than of the homogeneous solid component (white asterisk). Yellow arrowheads indicate low-signal-intensity rim on T2WI (A); the boundary between the tumour and myometrium appears well defined on the macroscopic image (B), and there is no fibrous tissue layer between the tumour and the myometrium on the microscopic image (C).

Furukawa et al reviewed three cases of low-grade ESS with intramyometrial mass patterns (the second pattern) and found that all the cases showed a low-signal-intensity rim on T2WI, with histologically confirmed fibrous tissue layers in two of the cases.^2^ Furukawa et al also suggested that the low-signal-intensity rim on MRI may reflect not only the fibrous tissue layer but also a decrease in free water due to a distortion of the myometrial tissue caused by tumour compression. This is because the pathologic fibrous tissue layer was thinner than its extent on MRI.^2^ In this case, the MRI showed a T2WI low-signal rim, while no corresponding fibrous tissue layer was observed on pathological examination (Figure 5), which is consistent with the characteristics of a low-grade ESS with an intramyometrial mass pattern. In addition, in contrast to a low-grade ESS, a leiomyoma (including a cellular leiomyoma) sometimes shows a T2WI high signal intensity rim called a pseudocapsule that reflects peri-tumoural oedema.^9^^,^^10^

In this case, the MRI findings showed that the mass consisted of three components, one of which showed a moderately high signal intensity on T2WI, a high signal intensity on DWI, and a low signal intensity on ADC, thus reflecting a high cell density. Typically, the presence of a solid component with a low ADC value in the mass is considered one of the characteristic MRI findings indicative of malignancy, such as a sarcoma or carcinoma due to a high cell density. However, even a cellular leiomyoma shows low ADC values reflecting its high cellularity.^3^ The ADC value of a low-grade ESS overlaps with that of cellular leiomyoma; therefore, it is difficult to differentiate them based solely on ADC values.^3^ Additionally, the solid component of a low-grade ESS often appears relatively homogeneous, without the speckled appearance, commonly observed in typical leiomyomas.^4^ The homogeneous solid component, along with the frequent occurrence of cystic degeneration, as demonstrated in this case, can be a valuable clue in the differential diagnosis of a low-grade ESS.^4^

On MRI, one of the three components of the mass showed a low signal intensity on T2WI, comparable to that of a muscle, and a low signal on DWI. Koyama et al reported that low-signal-intensity bands were observed on T2WI at the site of invasion of the myometrium in a low-grade ESS, a characteristic finding of a low-grade ESS. These T2WI low-signal bands corresponded to bundles of myometrium that had escaped invasion on pathologic examination.^8^ On comparing the MRI and pathological findings in this case, the low-signal-intensity structure on T2WI demonstrated a higher proportion of residual myometrium within the tumour (Figure 5), consistent with the previous reports.^8^

Generally, the presence of haemorrhage is often considered a factor for uterine malignancy; however, this does not apply to a low-grade ESS. A low-grade ESS is less frequently associated with haemorrhage compared to a high-grade ESS.^11^^,^^12^ In this case, the initial MRI showed a little haemorrhage, which may have been a factor in the misdiagnosis as a leiomyoma. Radiologists should realize that the absence of haemorrhage does not rule out the possibility of a low-grade ESS.

Other differential diagnoses include STUMP and leiomyosarcoma. The imaging findings of STUMP vary from “resembling leiomyoma” to “resembling leiomyosarcoma”. Typical leiomyosarcoma have irregular borders and show intratumoural haemorrhage and necrosis.^9^^,^^10^

In conclusion, this was an educational case in which the characteristic MRI findings made it possible to make a preoperative diagnosis of a low-grade ESS in the myometrium. The MRI findings, an intramyometrial mass with a low-signal-intensity rim on T2WI, a homogeneous solid component with mild hyperintensity on T2WI and low ADC values within the mass, a cystic component, and a low-signal-intensity component on T2WI similar to the muscle were consistent with previous reports.^2–4^^,^^8^ These MRI findings can be helpful to distinguish a low-grade ESS from a cellular leiomyoma. This case was appropriately diagnosed by focusing on MRI findings characteristic of a low-grade ESS.

## Learning points

- A low-grade ESS can present in three major MRI patterns: progression from the endometrium to myometrium (widely known), a well-defined mass in the myometrium (less well-known), and a polypoid mass in the uterine cavity (rare).
- A low-grade ESS located in the myometrium may mimic cellular leiomyoma on MRI. Since the treatment of a low-grade ESS is different from that of a cellular leiomyoma, it is important to differentiate between a low-grade ESS and uterine leiomyoma on MRI.
- The following MRI findings are the characteristics of a low-grade ESS located in the myometrium: a low-signal-intensity rim on T2WI, a homogeneous solid component with mild hyperintensity on T2WI, a cystic component, and a component with low signal intensity on T2WI similar to that of a muscle.
